# Reproductive Health Voucher Program and Facility Based Delivery in Informal Settlements in Nairobi: A Longitudinal Analysis

**DOI:** 10.1371/journal.pone.0080582

**Published:** 2013-11-18

**Authors:** Djesika D. Amendah, Martin Kavao Mutua, Catherine Kyobutungi, Evans Buliva, Ben Bellows

**Affiliations:** 1 African Population and Health Research Center, Nairobi, Kenya; 2 Jomo Kenyatta University of Agriculture and Technology, Nairobi, Kenya; 3 Population Council, Nairobi, Kenya; University of Alabama at Birmingham, United States of America

## Abstract

**Introduction:**

In Kenya, the maternal mortality rate had ranged from 328 to 501 deaths per 100,000 live births over the last three decades. To reduce these rates, the government launched in 2006 a means-tested reproductive health output-based approach (OBA) voucher program that covers costs of antenatal care, a facility-based delivery (FBD) and a postnatal visit in prequalified healthcare facilities. This paper investigated whether women who bought the voucher for their index child and had a FBD were more likely to deliver a subsequent child in a facility compared to those who did not buy vouchers.

**Methods and Findings:**

We used population-based cohort data from two Nairobi slums where the voucher program was piloted. We selected mothers of at least two children born between 2006 and 2012 and divided the mothers into two groups: Index-OBA mothers bought the voucher for the index child (N=352), and non-OBA mothers did not buy the voucher during the study period (N=514). The most complete model indicated that the adjusted odds-ratio of FBD of subsequent child when the index child was born in a facility was 3.89 (p<0.05) and 4.73 (p<0.01) in Group 2.

**Discussion and Conclusion:**

The study indicated that the voucher program improved poor women access to FBD. Furthermore, the FBD of an index child appeared to have a persistent effect, as a subsequent child of the same mother was more likely to be born in a facility as well. While women who purchased the voucher have higher odds of delivering their subsequent child in a facility, those odds were smaller than those of the women who did not buy the voucher. However, women who did not buy the voucher were less likely to deliver in a good healthcare facility, negating their possible benefit of facility-based deliveries. Pathways to improve access to FBD to all near poor women are needed.

## Introduction

In Kenya, the national maternal and neonatal mortality rates had remained high over the last three decades. The national mortality rate ranged from 328 to 501 deaths per 100,000 live births in 2003 for the preceding decade. More recent estimates of 488 obtained in 2008 fell within that same range [1,2]. Similarly, the neonatal mortality rate was estimated to be in the range of 31- 33 deaths per 1,000 live births for the last two decades [1]. Maternal mortality rates were even higher in some localities: in 2005 in Korogocho and Viwandani, two informal settlements in Nairobi, Kenya’s capital city, estimates of maternal mortality were 706 per 100,000 live births [3]. An estimated 60% of Nairobi residents lived in slums or slum-like conditions characterized by inadequate supply of government health services, education, and vital registration services among others. Then, it was not surprising that health indicators of slums dwellers were worse than the city average [4] or even the national average. Specifically, high rates of maternal and neonatal mortality were associated with poor access to antenatal care, failure to deliver in health facility or to seek medical assistance at delivery [2] all important features of the informal settlements [5,6].

To reduce the country’s high maternal and child mortality rates in accordance with the Millennium Development Goals, the government of Kenya with funding from the German Development Bank (KfW) launched in 2006 a reproductive health voucher program using an output-based approach (RH-OBA). The RH-OBA program currently operates in Korogocho and Viwandani, the two informal settlements of Nairobi mentioned above and four rural counties. The RH-OBA voucher uses a demand side approach by selling vouchers at nominal fee to low-income women who qualify on their poverty status. Women who purchased the vouchers, in turn, used them to access subsidized healthcare services in accredited public, private or faith-based health facilities of their choice [7]. The idea was that subsidizing facility-based deliveries would increase their number and thereby decrease maternal and neonatal mortalities especially in underserved areas of informal settlements and rural areas. 

A facility was accredited if it met criteria set by the public authorities in terms of staffing and quality of care. Limiting the choice of facility to accredited ones helped ensure acceptable quality of care as many facilities operating in the slums provide sub-standard care [5]. Providers were reimbursed at agreed rates that were intended to cover service delivery costs. More than 40 similar maternal and sexual health voucher programs had been implemented in other developing countries since the 1960s with growing interest in the past 15 years [8]. Two systematic reviews of health voucher programs found strong evidence that these programs could increase utilization of reproductive health goods and services. The reviews also found modest evidence that voucher programs could effectively target specific populations while improving the quality of services provided [7,9]. Health voucher programs might also help foster a stronger market for private or public health providers in underserved areas although further work was needed to describe the market [7,9]. 

The Kenya RH-OBA program sold safe motherhood and long-term family planning vouchers in targeted communities but gave a gender-based violence voucher free of charge at the facility to incentivize integrated service delivery, psychosocial support and legal services [10]. This study focused on the safe motherhood component, which was means-tested. Pregnant women who scored sufficiently low on a 14-item scale including housing characteristics, water source and sanitation, existing access to healthcare, and income were eligible to buy vouchers that entitled them to receive a package of care from their choice of accredited public and private facilities of the program. The RH-OBA voucher covered access to four antenatal care visits, a facility-based delivery (FBD) including treatment of complications if any, and a postnatal visit at the mother’s choice of qualified health facility. The cost of the safe motherhood voucher to the user was Kenya Shillings (KES) 200 (~USD 2.50). 

A previous study based on cross-sectional datasets from those two informal settlements reported that in 2006–08, women who used the voucher almost universally attended at least one antenatal visit (99.5%), and 96% of the women who used the voucher actually delivered in a facility. Among women who did not use the voucher, 94% received antenatal care and 61% delivered in a facility [11]. The national proportions for antenatal visit and facility based delivery were respectively 92% and 44% in 2008-09 [1]. While that study was the first to indicate a success of the RH-OBA approach to increasing facility-based deliveries in the African region, little was known on the longitudinal effect of the Kenya RH-OBA program on individuals’ observed location preferences for subsequent deliveries. 

This study seeked to answer the question: “Were women who bought the RH-OBA voucher for their index child, and delivered in a facility, more likely to deliver a subsequent child in a facility compared to women who did not buy the voucher during the period of the study?”

## Methods

### Study Setting and Dataset

This study data came from Korogocho and Viwandani, two informal settlements where the African Population and Health Research Center (APHRC) has been running the Nairobi Urban Health and Demographic Surveillance System (NUHDSS) since 2003. Each settlement covers about one kilometer square and is located within five to 10 kilometers from the city center. The NUHDSS records demographic events (births, deaths and migration) every four months and detailed household expenditures data once a year. The Viwandani informal settlement neighbors the Industrial Area of Nairobi and was a magnet for young males and relatively educated migrants in search of work while Korogocho was home for more settled families[12], some of whom had been living there for multiple generations. As a corollary of the different demographics in both settlements, households sizes were bigger in Korogocho than Viwandani while household income per capita was higher in Viwandani [13]. As of end of 2011, the latest data available showed that 32,746 households with 83,484 individuals lived in the area covered by the NUHDSS. This surveillance system provided vital statistics and other information on a population for whom these data would otherwise be unavailable. Nested within NUHDSS was the Maternal and Child Health (MCH) project, which recruited cohorts of mother-child pairs and followed them up every four months. A mother-child pair was recruited if the mother resided in the slum when pregnant and the child was 6 months old or younger at the time of recruitment. The MCH study covered the years 2006 to 2010 with a couple of recruitment suspensions between June and September 2009 and February to June 2010. During recruitment suspensions, follow-up interviews of existing cohorts were conducted. The INDEPTH (International Network for the Demographic Evaluation of Populations and Their Health) vaccination project (IVP) succeeded the MCH project from 2011 and took over existing cohorts of children while recruiting all children born from 2010 when recruitment into the MCH project ended. That strategy allowed for continuity in the recruitment and follow-up of children born in the slums since 2006 in the ongoing cohort studies. Both MCH and IVP projects were run by the same institution and team using the similar procedures and questionnaires so that the data quality was similar across all the rounds of data collections. 

The MCH/IVP projects collected information on the child’s place of delivery during the recruitment interview but the information on RH-OBA knowledge and use was collected at the second interview. Hence, information on the RH-OBA voucher was unavailable for mothers who dropped out of the MCH/IVP projects after the first recruitment interview, 22% in the MCH project and 27% in the INDEPTH Vaccination Project. 

This study included mother-children pairs from the MCH and the IVP datasets from 2006 to 2012. We selected mothers of at least two children, and for whom no important information—like the date of birth of the child or the mother—was missing or implausible. For twin births, we kept only one record knowing the mother’s information was the same and assuming both children were born in the same place. Since this paper focused on longitudinal effect of the voucher, we excluded women who did not buy the voucher for the index child but for the subsequent child.

The APHRC owns the datasets used in this analysis. APHRC has a data sharing policy that enables other researchers to access this dataset and others. APHRC data sharing policy is available at http://www.aphrc.org/insidepage/page.php?app=data

## Variables

We created three main binary variables for this study.

1. Facility-Based Delivery (FBD) =1 if the child was born in a health facility
2. Index child=1 if the child was the first one of that mother recruited in the MCH/IVP study, regardless of the mother’s parity
3. Subsequent child =1 if the child was a younger sibling of the index child from the same mother

We created two subgroups depending on whether the mother purchased a RH-OBA voucher. Group 1 included mothers who bought the RH-OBA when pregnant with the index child. Some mothers in Group 1 bought the vouchers for the subsequent child as well. Group 2 included mothers who did not buy the RH-OBA voucher during the study period. 

We created other independent variables as well. Maternal education was defined as the highest level attained by the mothers and distributed in three categories: 1) never attended school or did not finish primary school, 2) finished primary school, and 3) secondary or higher education levels. Parity at the time of the birth of the index or subsequent child was the number of children, dead or alive, born to the mother. The site where the household lived was a binary with Korogocho=1. We created also a quality of the facility variable. A facility was deemed good if it was among the 12 accredited by the voucher management agency, belonged to the government, or was operated by a reputable non-governmental or faith-based organization. In contrast, the quality in the other facilities was either unknown or bad. In any case, few healthcare facilities in the slums were able to provide objective quality of care measures aside from their recognized accreditation or affiliation [6,14].

We created a per capita expenditure variable at household level using household expenditures on food and non-food items (utility, health care, education, etc.) collected in the NUHDSS and adjusted by the size of the household. Children were assumed to consume half an adult’s expenditures. We merged both datasets and selected the information on household expenditures and assets in the year of birth of the child. We imputed the missing variables for the household expenditures per capita using site, village, previous and subsequent per capita expenditures of the household, household size, education level, ethnicity, age, duration of stay in the site of the household head. We used the Stata add-in for Imputation by Chained Equations (ICE) command in Stata 12, set the number of imputation to 5 and average the obtained values. This method of imputing missing values was applied to a cross-section data on the voucher program by some of the same authors [11]

Previous analyses indicated that the higher the parity, the less likely the mother was to use voucher for FBD [11], residents of Korogocho tended to have worse health outcomes than those in Viwandani [15] and FBD even among those who bought the voucher depended on their purchasing power, approximated here by the household per capita expenditures. 

### Empirical Analyses

The analytical dataset included 352 Index-OBA mothers, and 514 Non-OBA mothers. We first conducted descriptive analyses of all dependent and independent variables to gain an understanding of the main features of the dataset. We calculated unadjusted odds of FBD of the subsequent child if the index child was delivered in a facility. We then calculated adjusted odds-ratio with two models. Model 1 controlled for the specific settlements, maternal age, parity, year of birth of the subsequent child, whether the mother was in union, her education, and Model 2 included additionally the monthly household per-capita expenditures. We used Stata12 SE (State College) to merge the datasets and run the analyses. 

## Results

### Descriptive Statistics

Table 1 indicated that there were no systematic differences in the individual characteristics of mothers’ education, parity and age across the subgroups. Women in both groups were similar in their low education levels: 34% to 41% of mothers had no formal education or had not finished primary school. The women were also similar in the upper level as 18% of them in both groups had some secondary level of education or beyond. Parity at the birth of the index child was also similar across the subgroups ranging from 2.28 to 2.42. Although, mothers who did not buy the voucher appeared slightly older than those who did, the difference was not statistically different. 

**Table 1:** Descriptive statistics of main variables.

**Variables**	Group 1: Mothers bought OBA vouchers for index child		Group 2: Mothers did not buy vouchers at all during the study	
	N	Mean (Standard error)	N	Mean (Standard error)
RH-OBA used at index child delivery	352	0.78 (0.41)		
RH-OBA bought at subsequent child delivery	288	0.54 (0.50)		
RH-OBA used at subsequent child delivery	288	0.52 (0.50)		
FBD of index child	352	0.92*** (0.01)	514	0.64 (0.02)
FBD of subsequent child	352	0.84*** (0.02)	514	0.71 (0.02)
Quality of facility at index child birth	352	0.90*** (0.02)	514	0.53 (0.02)
Quality of facility at subsequent child birth	352	0.82*** (0.02)	514	0.63 (0.02)
No formal education / did not finish primary	352	0.39 (0.03)	514	0.34 (0.02)
Completed primary	352	0.43 (0.03)	514	0.47 (0.02)
Secondary school or more	352	0.18 (0.02)	514	0.18 (0.02)
Parity at index child birth	352	2.28 (0.09)	514	2.42 (0.08)
Maternal age	352	23.66 (0.28)	514	24.20 (0.24)
Mother in union	352	0.91*** (0.02)	514	0.96 (0.01)
Birth year index child	352	2007.57** (0.07)	514	2007.77 (0.06)
Korogocho	352	0.67*** (0.03)	514	0.55 (0.02)
Monthly household expenditures per capita at index child birth	298	2464.05** (69.76)	399	2740.13 (83.17)
Monthly household expenditures per capita at subsequent child birth	261	2784.82 (86.37)	366	2752.41 (84.22)
Imputed monthly household expenditures per capita during year of index child birth	343	2523.69** (62.55)	494	2765.46 (67.01)
Imputed monthly household expenditures per capita during year of subsequent child birth	350	2890.18 (69.62)	504	2929.59 (71.26)

*** p<0.01, ** p<0.05, * p<0.1 difference in the subgroups of mothers who bought the voucher for the index- OBA child and those who did not buy vouchers at all (Group 1: index-OBA versus Group 2:non-OBA)

The variable In-Union indicated that mothers who bought the voucher for their index children were less likely to be in union than mothers who did not. The main difference across the groups related to poverty and place of residence. 

The monthly household per capita expenditures were captured at two different points in time: during the years of the birth of the index and the subsequent children. Initially, the OBA mothers had a per-capita household expenditures of KES 2464 (USD 30.80) which were KES 276 (USD 3.45) lower than the monthly expenditures of KES 2740 (USD 34.25) of the non-OBA mothers (p<0.05). Conversely, the OBA mothers had slightly higher household expenditures (> KES 32) during the year of the birth of the subsequent child but the difference was not statistically significant. Results using imputed values for the missing household expenditures indicate a similar pattern: OBA mothers had significantly lower household expenditures during the index child year of birth but not when the subsequent child was born. 

The majority of women in the dataset come from Korogocho. Interestingly, six children out of 10 were born in a facility in both groups. However, the trend was mixed: OBA mothers had FBD proportions of 92% for their index child, but only 84% of the subsequent ones. On the contrary, non-OBA mother had FBD proportions of 64% and 71% respectively, which indicated an increasing secular trend toward FBD.

The quality of facilities where women delivered followed a trend similar to the FBD: 90% of OBA mothers attended a good quality facility for the index child but only 82% did for the subsequent child. Conversely, those proportions were 53% and 63% among non-OBA mothers. 

Only 78% of women who bought the RH-OBA voucher for the index child used it for his/her delivery, leaving an open question on the place of delivery of the 22% who did not. Furthermore, only 54% of women who bought the voucher for the index child did so for the subsequent child as well. Among them, 94% had a FBD. In contrast, among women who did not buy the voucher for the subsequent child, only 72% delivered in a facility (p<0.01). No statistical difference was found in the per-capita expenditures or education level of the mothers who bought again the voucher or those who did not. 

### Unadjusted odds ratio

In both groups, the odds that a subsequent child was born in a facility if the index child was also born in a facility were higher (Table 2). The numbers were 3.68 (p<0.01) for the index-OBA, and 4.43 (p<0.01) non-OBA groups respectively. When mothers had three children in the dataset, the odds that the youngest child was born in a facility were also higher than that of the index children. The odds of FBD of the third child was 8.5 (p<0.01) among index-OBA mothers but only 3.43 (p<0.1) among non-OBA mothers. 

**Table 2:** Crude Odds Ratios for a facility based delivery (FBD) of the subsequent children by voucher purchase status.

VARIABLES	Mother bought the RH-OBA while pregnant of the index child		Mother did not buy the RH-OBA voucher	
	FBD of subsequent child	FBD of the child rank 2 following the index child	FBD of subsequent child	FBD of the child rank 2 following the index child
	(1)	(2)	(4)	(5)
FBD of index child	3.68***		4.43***	
	(1.53)		(0.91)	
Constant	1.64		1.06	
	(0.63)		(0.16)	
FBD of index child		8.50**		3.43*
		(8.27)		(2.40)
Constant		1		0.88
		(0.82)		(0.45)
Observations	352	44	514	39

Standard error in parentheses*** p<0.01, ** p<0.05, * p<0.1

### Adjusted odds ratio

Table 3 indicated that among the OBA mothers, the odds of FBD delivery of the subsequent child given that the index child was born in a facility was 2.98 (p<0.05) for Model 1 controlling only for maternal characteristics and 3.89 (p<0.05) for Model 2, which added household expenditures per capita. Among the non-OBA mothers, the adjusted odds-ratio were 4.28 (p<0.01) for Model 1 and 4.73 (p<0.01) for Model 2. 

**Table 3:** Odds ratio and (Standard deviation) of facility-based delivery (FBD) of the subsequent children.

VARIABLES	FBD of subsequent child			
	Mother bought the RH-OBA for the index child		Mother did not buy the RH-OBA voucher	
	Model 1	Model 2	Model 1	Model 2
FBD of index child (reference no FBD)	2.98**	3.89**	4.28***	4.73***
	(1.45)	(2.14)	(0.93)	(1.20)
Korogocho (reference: Viwandani)	4.23***	5.72***	2.60***	2.70***
	(1.43)	(2.36)	(0.61)	(0.75)
Maternal Age	1.04	0.99	1.05	1.06
	(0.050)	(0.06)	(0.03)	(0.04)
year of delivery of subsequent child	1.68***	2.13***	1.27***	1.33**
	(0.22)	(0.40)	(0.10)	(0.15)
Parity	1.03	1.23	0.94	0.87
	(0.15)	(0.22)	(0.01)	(0.11)
Union (reference: mother not in union)	1.86	1.14	2.76**	2.56
	(0.97)	(0.82)	(1.33)	(1.48)
Mother finished primary school (reference: never attended/did not finish primary school)	1.11	1.37	0.83	0.89
	(0.41)	(0.61)	(0.21)	(0.26)
Mother attended secondary school or more (reference: never attended/did not finish primary school)	1.45	1.40	1.41	1.75
	(0.73)	(0.81)	(0.51)	(0.74)
Household expenditures per capita in year of delivery of subsequent child		1.00		1.00
		(0.00)		(0.00)
Observations	352	261	514	366

Standard error in parentheses*** p<0.01, ** p<0.05, * p<0.1

For both models and groups, the year of delivery was statistically significant suggesting a secular trend toward FBD other factors held constant. The odds of FBD were also higher if the mother lived in Korogocho. Other variables were not significant in terms of either magnitude or statistics. Interestingly, while the expenditures per capita variable itself was not significant, its inclusion in the regression improved the precision of the odds-ratio of the main variables. We ran the again the analyses using the imputed values for household expenditures when those were missing. The results were similar to those obtained without imputation in terms of magnitude of the coefficients and statistical significance (not shown).

### Discussion and Limitations

This study found that women who had an index child in a facility were more likely to have a subsequent child also in a facility. However, the odds of FBD were smaller among women who bought the voucher for their index child, compared to those who did not. A higher proportion index-OBA mothers had a subsequent child in a facility compared to non-OBA mothers.

Surprisingly, only 54% of the women who bought the voucher for the index child also bought it for the subsequent one. Many reasons might explain this fact. First, the OBA mothers might not have been poor anymore. Second, they might have decided against further participation in the program because of cumbersome checks of eligibility criteria. Finally, the OBA mothers might have given birth again during the year where no new voucher was issued as the stakeholders were in court over management dispute. In any case, the fact that those who bought again the voucher were significantly more likely to deliver in a facility corroborated the positive correlation between access to the OBA voucher and FBD. 

Note that not all FBD were equal. Women who used the voucher for FBD had to attend accredited facilities, so they probably received appropriate quality care. The same cannot be ascertained about women in the non-OBA group who could deliver in non-accredited facilities. The importance of accredited place of delivery should be emphasized as a previous study showed that although two third of women in Korogocho and Viwandani had delivered in a facility, fewer than half of them were assisted by a skilled birth attendant [5] who could prevent, diagnose or treat complications. Moreover, only 2 out of 25 facilities in those informal settlements could provide emergency obstetric quality care [14]. 

Another difference between the index-OBA and non-OBA groups related to poverty. Mothers who bought the voucher had statistically lower household per capita expenditures than mothers who did (p<0.05) during the year of birth of their index child. However, there was no statistical difference between the two groups of mothers at the birth of the subsequent child. We speculate that the absence of difference may be an artifact of the small sample size. 

This study observed a secular trend of increasing facility-based deliveries among residents of Korogocho and Viwandani, similar to the previous findings. Nevertheless, among the non-OBA mothers, a substantial proportion of mothers did not have a FBD. A couple of qualitative studies conducted in the same slums in 2005 and 2006 just before the start of the voucher project found that poverty was one of the key factors behind homebirths. Study respondents including women who had a previous life-threatening birthing experience, their partners and other opinion leaders indicated that costs were a major impediment to seeking emergency obstetric care[16,17]. Those costs ranged from twenty KES (USD 0.20) for registration in a government hospital to KES 5,500 (USD=68.7) in a mission hospital for normal delivery. For caesarian section, the costs ranged from KES 3000 to 30,000 (USD=37.5 to 375). Transportation cost varying from KES 3,000 to 4,000 (37.5 to 50) should be added to the direct medical costs. These estimated costs of emergency care from patients’ perspective should be compared with the average expenditures per capita that did not exceed KES 2,784 in any group in this study. Respondents submitted that FBD were associated with such high costs that it should be considered only as the last recourse. Thus, pregnant women attend antenatal care to prepare for homebirth and to avoid FBD. The voucher program addressed partly the monetary obstacle by subsidizing the cost of care and removing the need to pay at the point of delivery. 

Respondents in both qualitative surveys also pointed out other non-monetary reasons to avoid FBD such as the unfriendly/disrespectful attitude of providers toward them, poor physical access to hospital etc. To address the non-monetary issues, prequalified providers received an output-based reimbursement, thus tying the quality of their service to their financial rewards. Providers who offer quality care in a friendly manner were more likely to attract patients and reap the benefit of the program.

This paper presented some limitations, which suggests caution in the interpretation of the results and the policy recommendations. The first one was the small sample sizes of the study. Larger sample size would probably reduce standard errors and improve the statistical significance of the other variables in the models lending strength to the results. A second limitation was the measurement of the socio-economic status of the mother. We approximated it with per-capita household expenditures. This variable was not collected in the IVP survey but in the NUHDSS and only once a year. The expenditures during the year of birth of the child used in this study might fail to capture the household’s standing during of pregnancy. Compounding the issue was the missing values of the expenditures for at least 15% of the sample. We used imputed values and obtained similar results. Other explorations using an asset index or income per capita collected at the same time as the household assets gave similar results suggesting their robustness. 

In addition, recruitment in the study was suspended twice for about three months each time, but those suspensions were unlikely to bias substantially the result of this study since they were limited in time and no significant event occurred then. 

Despite issues of sample size, the study has a number of strengths. First, it was population-based cohort data and as such presents reduced instances of sampling bias. In addition, the data include a cohort of mothers with multiple births lending consistency to the information collected. Finally, information related to each child was collected before another was born, reducing the risk of recall bias or confusion between specific place of birth or OBA use for each child. 

## Conclusions

The study indicated that the voucher program improved poor women access to FBD. Furthermore, the FBD of an index child appeared to have a persistent effect, as a subsequent child of the same mother was more likely to be born in a facility as well. While women who purchased the voucher have higher odds of delivering their subsequent child in a facility, those odds were smaller than those of the women who did not buy the voucher. Note, however, that women who did not buy the voucher were also less likely to deliver in a good healthcare facility, which negated their possible benefit of facility-based deliveries. Additional pathways to improve access to FBD to all near poor women are needed. 

## References

[1] Kenya National Bureau of Statistics, ICF Macro (2010) Kenya Demographic and Health Survey 2008-09. Calverton, Maryland: KNBS and ICF Macro.

[2] Central Bureau of Statistics (CBS) [Kenya] MoHMK, and ORC Macro (2004) Kenya Demographic and Health Survey 2003. Calverton, MD: CBS, MOH, and ORC Macro.

[3] Ziraba AK, Madise N, Mills S, Kyobutungi C, Ezeh A (2009) Maternal mortality in the informal settlements of Nairobi city: what do we know? Reprod Health 6: 6. doi:10.1186/1742-4755-6-6. PubMed: 19386134. 19386134 PMC2675520

[4] African Population and Health Research Center; (2002) Population and Health Dynamics in Nairobi's Informal Settlements: Report of the Nairobi Cross-sectional Slums Survey (NCSS) 2000. Nairobi: African Population and Health Research Center.

[5] Fotso JC, Ezeh A, Madise N, Ziraba A, Ogollah R (2009) What does access to maternal care mean among the urban poor? Factors associated with use of appropriate maternal health services in the slum settlements of Nairobi, Kenya. Matern Child Health J 13: 130-137. doi:10.1007/s10995-008-0326-4. PubMed: 18297380. 18297380

[6] Fotso JC, Ezeh A, Oronje R (2008) Provision and Use of Maternal Health Servies among Urban Poor Women in Kenya: What Do We Know and What Can We Do? Bulletin of The New York Academy of Medecine 85: 428-442. 10.1007/s11524-008-9263-1 PMC2329740 18389376

[7] Bellows NM, Bellows BW, Warren C (2011) Systematic Review: the use of vouchers for reproductive health services in developing countries: systematic review. Trop Med Int Health 16: 84-96. doi:10.1111/j.1365-3156.2010.02667.x. PubMed: 21044235. 21044235

[8] Gorter A, Grainger C, Okal J, Bellows B (2012) Lessons from sexual and reproductive health voucher program design and function: A systematic review. IUSSP Scientific Panel on Reproductive Health. Bangkok.

[9] Meyer C, Bellows NM, Campbell M, Potts M (2011) The Impact of Vouchers on the Use and Quality of Health Goods and Services in Developing Countries: A systematic review. London: EPPI-Centre. Social Science Research Unit, Institute of Education, University of London.

[10] Njuki R, Okal J, Warren CE, Obare F, Abuya T et al. (2012) Exploring the effectiveness of the output-based aid voucher program to increase uptake of gender-based violence recovery services in Kenya: a qualitative evaluation. BMC Public Health 12: 426. doi:10.1186/1471-2458-12-426. PubMed: 22691436. 22691436 PMC3413608

[11] Bellows B, Kyobutungi C, Mutua MK, Warren C, Ezeh A (2013) Increase in facility-based deliveries associated with a maternal health voucher programme in informal settlements in Nairobi, Kenya. Health Policy Plan Mar 28: 134-142. 10.1093/heapol/czs030 PMC3584990 22437506

[12] Fotso J-C, Ezeh AC, Essendi H (2009) Maternal health in resource-poor urban settings: how does women's autonomy influence the utilization of obstetric care services? Reprod Health 6: 9-. PubMed: 19531235. 19531235 10.1186/1742-4755-6-9 PMC2706794

[13] Emina J, Beguy D, Zulu EM, Ezeh AC, Muindi K et al. (2011) Monitoring of health and demographic outcomes in poor urban settlements: evidence from the Nairobi Urban Health and Demographic Surveillance System. J Urban Health 88 Suppl 2: S200-S218. doi:10.1007/s11524-011-9594-1. PubMed: 21713553. 21713553 PMC3132229

[14] Ziraba AK, Mills S, Madise N, Saliku T, Fotso JC (2009) The state of emergency obstetric care services in Nairobi informal settlements and environs: results from a maternity health facility survey. BMC Health Serv Res 9: 46. doi:10.1186/1472-6963-9-46. PubMed: 19284626. 19284626 PMC2662828

[15] African Population and Health Research Center; (2012) Indicators from NUDHSS , 2003-2011. Nairobi: APHRC.

[16] Essendi H, Mills S, Fotso JC (2011) Barriers to formal emergency obstetric care services' utilization. J Urban Health 88 Suppl 2: S356-S369. doi:10.1007/s11524-010-9481-1. PubMed: 20700769. 20700769 PMC3132235

[17] Izugbara CO, Kabiru CW, Zulu EM (2009) Urban poor kenyan women and hospital-based delivery. Public Health Rep 124: 585–589. PubMed: 19618796. 19618796 10.1177/003335490912400416 PMC2693173

